# Transoral balloon kyphoplasty in a myeloma patient with painful osseous destruction of the corpus vertebrae axis

**DOI:** 10.1093/jscr/rjae009

**Published:** 2024-01-30

**Authors:** Julian Ramin Andresen, Harald Widhalm, Reimer Andresen

**Affiliations:** Department of Orthopedics and Trauma Surgery, Medical University of Vienna, Vienna AT-1090, Austria; Department of Orthopedics and Trauma Surgery, Medical University of Vienna, Vienna AT-1090, Austria; Institute of Diagnostic and Interventional Radiology/Neuroradiology, Westkuestenklinikum Heide, Academic Teaching Hospital of the Universities of Kiel, Luebeck and Hamburg, Heide DE-25746, Germany

**Keywords:** transoral balloon kyphoplasty, interventional pain management, quality of life, multiple myeloma, osseous destruction, pathological fracture

## Abstract

Multiple myeloma is the most common primary malignant disease of the spine, which can lead to pathological fractures with consecutive instability and immobilizing pain, due to osseous destruction of individual vertebral bodies. The different surgical care is challenging, although good stabilization should be achieved if possible. The resulting blocking of micro-movements leads to pain minimization. However, this is a symptomatic therapy and does not address the primary disease. In the following, we report on successful transoral balloon kyphoplasty for the treatment of myeloma-related osteolysis with a pathological fracture of vertebral body C2, which led to a significant clinical improvement.

## Introduction

Multiple myeloma (MM) is a malignant disease characterized by monoclonal proliferation of plasma cells in the bone marrow, with increased production of complete or incomplete monoclonal immunoglobulins. These are detectable in both serum and urine. The incidence is about 3–6 new cases/100 000 population per year, men being affected slightly more often than women, whilst the median age of onset is about 73 years. About 4200 patients die of MM in Germany every year. There is an increased risk in first-degree relatives, whilst other risk factors under discussion include exposure to ionizing radiation, pesticides or petrochemical products, as well as obesity and chronic infections [1, 2]. MM is regularly preceded by a monoclonal gammopathy of undetermined significance [3], and the progression to MM requiring treatment is ~1%/year [4].

Clinical symptoms are often non-specific, with anaemia, reduced performance, bone pain, hypercalcaemia syndrome, symptomatic renal insufficiency, weight loss, and a tendency to infection [5]. In rare cases, the first symptom is an immobilizing painful pathological fracture in the spine [6], whereby osteoloytic/osteopenic destruction is not uncommon in ~70% [7] of those affected and 60% of myeloma patients suffer a pathological fracture during the course of their disease [8]. Involvement of vertebral bodies C1 and C2 is quite rare; in this context, Barragan-Campos *et al.* [9] reported that in 117 patients with vertebral metastases, 45.3% of whom had breast cancer, 14.5% lung cancer, 7.7% MM, and 32.5% other tumour entities, the cervical spine was involved in 10% and vertebral body C2 in just 2%.

The diagnosis is rendered according to the criteria of the International Myeloma Working Group (IMWG) [5]. Beside laboratory tests, modern imaging plays a central role. For the detection of osseous destruction, low-dose whole-body computed tomography is now the standard [10], whereby destructive medullary infiltrates in the peripheral skeleton can also be reliably detected [11], thus replacing projection radiography [10, 12].^ 18^F-Fluordesoxyglucose positron emission tomography–computed tomography (^18^F-FDG PET/CT) and magnetic resonance imaging (MRI) with diffusion-weighted and strongly T2-weighted fat-suppressed sequences (STIR) are used as imaging in follow-up examinations, where they have prognostic relevance [10, 12]. An exact assignment of symptomatic vertebral body deformities is best achieved by the detection of oedema zones in the vertebral bodies in STIR-weighted MRI.

The initiation of therapy is indicated in symptomatic MM according to the criteria of the IMWG [5]. In suitable patients, high-dose chemotherapy followed by autologous stem cell therapy and maintenance therapy is favoured [13]. For patients who are not eligible for this, there is a variety of different combinations of chemotherapy. An additional improvement in response rates and progression-free survival can be achieved with monoclonal antibodies such as daratumumab (Darzalex^®^) [14]. Supportive osteoprotective therapy, e.g. with zoledronic acid (Zometa^®^) [15] or denosumab (Xgeva^®^), should always be carried out [16, 17].

In the presence of symptomatic pathological fractures and/or osseous destruction of the axial skeleton, cement augmentation by means of vertebroplasty [18, 19] or balloon kyphoplasty (BKP) [20–22] has already proven effective for rapid pain reduction, and both procedures were included in a consensus statement from the IMWG [23]. In order to achieve sufficient stabilization, BKP is sometimes performed in combination with screw osteosynthesis [24] or spondylodesis [25].

In contrast to other surgical stabilization operations, there are only a few cases of transoral kyphoplasty of the corpus vertebrae axis in the literature. The technical feasibility, safety and clinical outcome of a patient with painful myeloma-related osteolysis in vertebral body C2 are reported below.

## Case report

A 68-year-old patient with painful, myeloma-related osseous destruction of the corpus vertebrae axis underwent transoral BKP. Taking into account the Spinal Instability Neoplastic Score [26], 12 score points were found, which corresponds to the beginning of instability of the vertebral body C2 lesion. The indication for cement augmentation by means of BKP was made in an interdisciplinary case conference consisting of oncologists /internists, orthopaedists/trauma surgeons, neurosurgeons, and interventional radiologists, whereby the highest priority was to reduce the immobilizing pain as quickly as possible.

### Technique and procedure

To detect tumour spread, whole-body computed tomography was performed using a low-dose technique (from the top of the skull to below the knee joints; axially in bone window and soft-tissue window with a slice thickness (ST) of 2.5 mm, as well as axial, coronal, and sagittal reformation with an ST of 1.25 mm) before the start of therapy. MRI of the axial skeleton (sagittal T1-, T2-weighted 4 mm, coronal STIR 4 mm and axial T1-, T2-weighted 2.8 mm slices) was also performed to detect symptomatic lesions. A conventional X-ray of the cervical spine in 2 planes in the standing position was performed to detect vertebral body sintering or axial deviation before the intervention and for follow-up assessment on the day of inpatient discharge.

The surgical procedure was performed by R.A.

**Figure 1:**
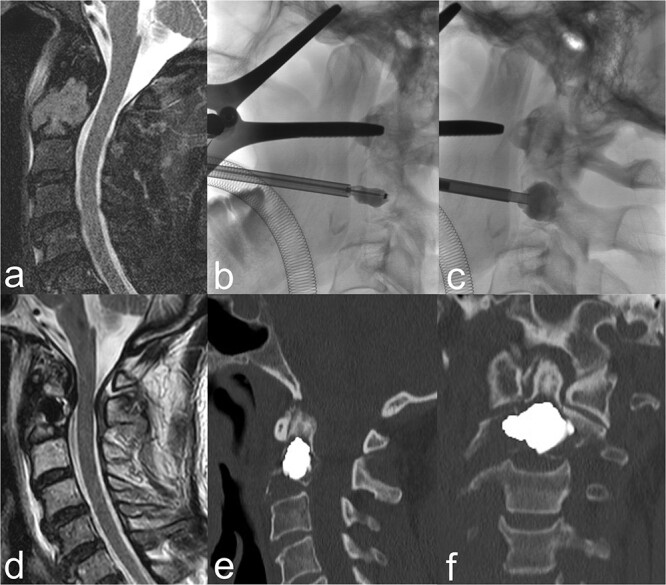
(a) The sagittal heavily T2-weighted fat-suppressed MRI imaging shows myeloma-related osseous destruction/pathological fracture of the corpus vertebrae axis. (b) BKP via a transoral approach. (c) After removal of the balloon catheter, the cavity created is filled with PMMA cement. (d) Centrally located cement plug in the sagittal T2-weighted MRI slice. (e) and (f) The sagittal and coronal reformed CT slices show a cement plug located centrally in the lesion. Cement leakage can be ruled out.

As a matter of routine, perioperative prophylactic single-shot antibiotic treatment was given with 2 g cefazolin (Cefazolin HEXAL^®^ 2 g). The intervention was performed under intubation anaesthesia. The patient was then placed in the C-arm in the supine position with slight hyperextension of the cervical spine. The oropharynx was held open using a spreader. After the usual preparation and determination of the access plane, a Kirschner wire was first advanced transorally into the central tumour lesion of vertebral body C2. A 19-G hollow needle was then inserted via the wire. A 15 mm balloon catheter (Kyphon Xpander™ II system™) was introduced through this and inflated and deflated several times, partially overlapping, under fluoroscopy. The cavity prepared in this way was then successively filled with 2.6 ml of viscous PMMA cement (Kyphon VuE™ bone cement) using a low-pressure method, also under fluoroscopy (Fig. 1a–c).

### Course

To exclude cement leakage, a spiral CT in thin-slice technique with coronal and sagittal reformation in 2 mm ST was performed on postoperative day 1. The patient was remobilized without a collar from the 1st postoperative day on. Pain intensity was determined by visual analogue scale (VAS) before the intervention, on postoperative day 2 and 6 months after osteoplastic cement augmentation. Finally, the patient was asked to rate their satisfaction and change in quality of life.

### Outcome

**Figure 2:**
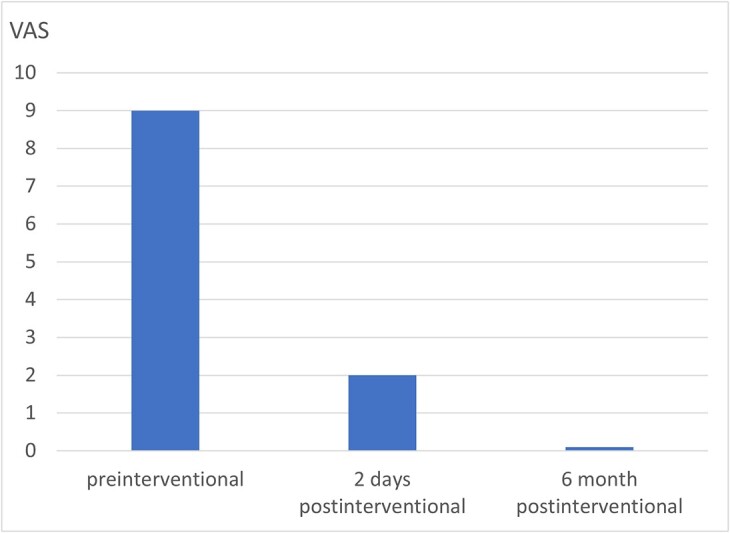
Pain development of the osseous destruction of the corpus vertebrae axis in the course after BKP.

BKP was technically fully feasible. The control CT showed a central cement distribution in the tumour lesion, whilst cement leakage could be excluded (Fig. 1d–f). A significant, rapid and sustained pain reduction developed from 9 score points on the VAS pre-intervention to 2 points on postoperative day 2 and complete freedom from pain under provoked movement after 6 months (Fig. 2). The patient was able to be quickly remobilised after the intervention and be passed on for the further planned therapeutic measures. Patient satisfaction with the procedure was high, whilst quality of life improved significantly subjectively and objectively. The patient would not hesitate to have the intervention performed again.

## Discussion

Successful interventional treatment of osseous destruction of vertebral body C2, via a transoral approach, has only been reported by individual working groups to date. The first cement augmentations for the treatment of a symptomatic angioma [27], an aneurysmal bone cyst [28], a metastasis from a thyroid carcinoma [29], and a multiple myeloma [30] were performed without relevant complications using the vertebroplasty technique, whereby a marked reduction in pain was achieved clinically. Monterumici *et al.* [31] were the first to perform transoral kyphoplasty in vertebral body C2 in three patients with osteolysis, one of whom was a 69-year-old woman with MM, with no complications and marked clinical improvement in terms of sustained pain reduction and fracture stabilization. BKP is an osteoplastic procedure in which a preformed cavity is created in the osseous lesion by inflation of a balloon for cement insertion, whilst the circumference is compressed, thus minimizing cement leakage [21, 22]. CT diagnostic imaging also ruled out leakage in our patient, with good central cement filling (Fig. 1e and f). In this respect, the risk of cement leakage would appear to be more likely with the vertebroplasty procedure [32].

To prevent infection in relation to transoral approach, preoperative disinfection of the pharyngeal mucosa, intravenous antibiotics and aseptic insertion of the instruments used are mandatory [28, 31]. An inflammation in the nasopharynx is a contraindication. Good planning of the access route is necessary to avoid further complications, although a transpedicular approach is not possible, as the pedicles are too small. A lateral, paravertebral approach seems very risky for vertebral body C2, due to the nerve structures and the vessels running in close proximity. A possible route that is chosen most frequently is the anterolateral from submandibular [22, 33]. Like other working groups [27–32], we consider that the transoral approach provides the best anatomical overview for cement augmentation to treat osseous destruction in vertebral body C2 and is thus the most reliable for avoiding injury to adjacent structures.

## Conclusion

Using a transoral approach, BKP is a safe and feasible procedure for treating a myeloma-related lesion in the C2 vertebral body segment. The cement augmentation may achieve, as in this case, a stabilization of the osseous destruction and consecutively a significant, rapid and sustained reduction in pain. Short-term mobilization is then possible, which considerably improves the feasibility of further therapies required and improves the overall quality of life.
